# Head Pose Estimation on Top of Haar-Like Face Detection: A Study Using the Kinect Sensor

**DOI:** 10.3390/s150920945

**Published:** 2015-08-26

**Authors:** Anwar Saeed, Ayoub Al-Hamadi, Ahmed Ghoneim

**Affiliations:** 1 Institute for Information Technology and Communications (IIKT), Otto-von-Guericke-University Magdeburg, Magdeburg D-39016, Germany; E-Mail: Ayoub.Al-Hamadi@ovgu.de; 2 Department of Software Engineering, College of Computer Science and Information Sciences, King Saud University, Riyadh 11451, Saudi Arabia; E-Mail: ghoneim@KSU.EDU.SA; 3 Department of Computer Science, College of Science, Menoufia University, Menoufia 32721, Egypt

**Keywords:** head pose, local binary pattern, histogram of gradient, Gabor filter, Kinect sensor, support vector machine, regression

## Abstract

Head pose estimation is a crucial initial task for human face analysis, which is employed in several computer vision systems, such as: facial expression recognition, head gesture recognition, yawn detection, *etc*. In this work, we propose a frame-based approach to estimate the head pose on top of the Viola and Jones (VJ) Haar-like face detector. Several appearance and depth-based feature types are employed for the pose estimation, where comparisons between them in terms of accuracy and speed are presented. It is clearly shown through this work that using the depth data, we improve the accuracy of the head pose estimation. Additionally, we can spot positive detections, faces in profile views detected by the frontal model, that are wrongly cropped due to background disturbances. We introduce a new depth-based feature descriptor that provides competitive estimation results with a lower computation time. Evaluation on a benchmark Kinect database shows that the histogram of oriented gradients and the developed depth-based features are more distinctive for the head pose estimation, where they compare favorably to the current state-of-the-art approaches. Using a concatenation of the aforementioned feature types, we achieved a head pose estimation with average errors not exceeding $5.1^{\circ },4.6^{\circ },4.2^{\circ }$ for pitch, yaw and roll angles, respectively.

## 1. Introduction

Head pose estimation is considered as the first step in several computer vision systems, such as: facial expression recognition, face recognition, head gesture recognition, gaze recognition, driver monitoring, *etc*. For example, many researchers adapt their approach to do multi-/cross-pose facial expression recognition: Niese *et al*. [1] propose a method that infers pose-invariant facial expression recognition from image sequences, where the pose dependency is removed by transforming the current estimated pose into a nearly frontal face to correct the calculated optical flow features. Considering different poses, Moore and Bowden [2] developed a texture-based approach to perform a multi-view facial expression recognition. By learning the mapping between facial points in each pair of discrete non-frontal poses and their corresponding frontal pose, Rudovic *et al*. [3] propose a coupled scaled Gaussian process regression (CSGPR) model for head pose normalization to perform pose-invariant facial expression recognition. In a similar way, a robust estimation of the head pose leads to pose-invariant face recognition [4]. A continuous estimation of the head pose over an image sequence is an essential task for head gesture recognition. Morency and Darrell [5] use the nod of the person’s head as the user interface commands, precisely for dialog box confirmation and document browsing. Head gestures are also considered as a language in human to robot conversations, where human can instruct the robot or give it feedback [6]. To read the human mental state, several modalities should be fused, and one of them is the head gesture [7,8]. Gaze direction can be inferred from the head pose [9], where an entire database is dedicated for this purpose [10]. Head pose provides rich information about the visual focus of attention, which is employed in different applications, such as: human behavior analysis in multi-person scenarios [11] and driver assistance systems [12,13].

**Figure 1:**
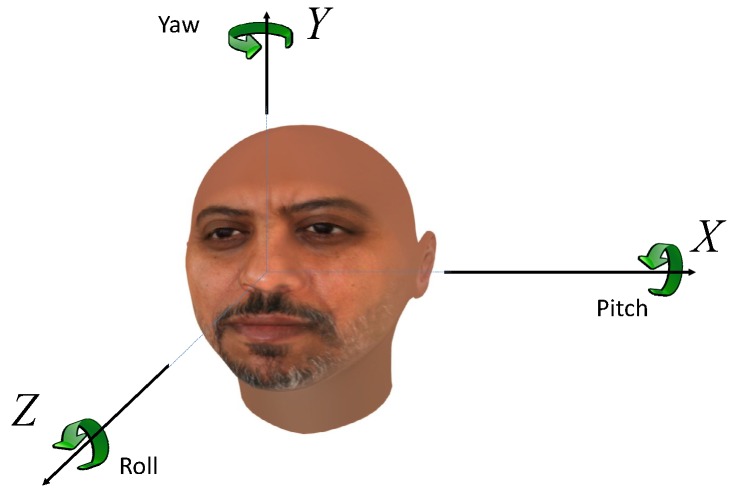
The head pose rotation angles. Yaw is the rotation around the *Y*-axis. Pitch around the *X*-axis, roll around the *Z*-axis.

In computer vision, face pose estimation is defined as the process of deducing the face orientation from a single image/a sequence of 2D/3D images. The face is usually modeled as a rigid object, with three DOF in the pose characterized by three rotation angles: pitch, roll and yaw. With a human head facing the camera, yaw is the angle of moving the head left and right (rotation around the *Y*-axis); the pitch is that of moving the head up and down (rotation around the *X*-axis); and roll is the tilt angle (rotation around the *Z*-axis); as shown in Figure 1.

Throughout the last two decades, a number of approaches was proposed to tackle the face pose estimation from 2D/3D facial data. Those approaches can be categorized according to several criteria, such as: temporal dependency, estimation continuity, data source, *etc*.

### 1.1. Temporal Dependency

Considering the temporal dependency criterion, frame-based approaches refer to those that infer the head pose from the considered frame data, in other words without employing any temporal information. Murphy-Chutorian *et al*. [12] estimate the head pose of each frame with the help of histogram of local gradients features extracted from a detected face patch. Similarly, other approaches use different texture features extracted from a single face patch: local Gabor binary pattern [14], Gabor filter [15] and histogram of gradients [16]. Gurbuz *et al*. [17] propose a model free approach to estimate the head pose using stereovision. They utilize the reconstructed face plane along with the eye locations to estimate each instance of pose. In contrast to frame-based methods, several approaches utilize the temporal information either to enhance the pose estimation accuracy or to estimate a wider range of head poses. Without previous training, Jimenez *et al*. [18] use a stereo camera to infer the current human head pose. In their proposed approach, a 3D face model is created from 2D points superposed over the frontal face image using a stereo correspondence. Then, random sample consensus (RANSAC) and pose from orthography and scaling with iterations (POSIT) algorithms are used to track the 2D points and then to deduce the human pose at each frame, assuming the tracking starts from a frontal pose of zero rotation angles. Otherwise, the initial angles will appear as a constant offset error. Tu *et al*. [19] approach tracks the head pose in low resolution videos with the help of a particle filtering framework, where the appearance variations are modeled online by the incremental weighted PCA subspace with a forgetting mechanism. Similar to many other approaches, they assume that the human face tracking starts with a detected face of zero rotation angles. Utilizing multiple cameras to enhance the facial point tracking is an incorporated option in several approaches. Ruddarraju *et al*. [20] extend an eye-tracking method from a single camera system to a multiple camera system, where the head pose is estimated by triangulating multiple facial features obtained from the eye tracker.

### 1.2. Data Source

Considering the input data source of the head pose approach, most aforementioned approaches estimate the head pose in gray/color 2D images. Some approaches enhance their face tracker with the help of 3D information stemming either from the use of stereo/multi-cameras or cameras with a depth sensing sensor. Nowadays, several approaches exploit the depth information offered by the consumer Kinect sensor. As opposed to the color image texture, the depth data are less sensitive to the illumination variations. Based only on the depth data, Niese *et al*. [21] create a person-specific head model that consists of 3D point vertices and surface normals. Then, they use the iterative closest point (ICP) algorithm to fit this head model to the current head pose of the considered person, assuming that the face is located in the upper part of the point cloud, and the rotation starts from smaller angle values. Fanelli *et al*. [22] also use only the depth data offered by the Kinect sensor to estimate the head pose. Their approach is based on discriminative random regression forests where each node splitting is supposed to reduce the entropy of the class label distribution and the variance of the head position and orientation. The employed random forests are supposed to detect the face patch, as well. With the help of features extracted from both the color and depth images of a Kinect sensor, Yang *et al*. [23] use three steps to arrive at an estimate of the head pose. First, they detect a coarse location of the face. Then, based on the coarse detection, they perform a refining search on the image coordinates and scales to find the accurate head location. Finally, they estimate the head pose with the help of a feed-forward multi-layer perceptron (MLP) network. Buddharaju *et al*. [24] propose an approach to estimate the head pose from thermal images.

### 1.3. Estimation Continuity (Pose Domain)

The proposed approaches can be categorized into two groups according to the domain of their pose estimate. The first group returns discrete pose estimates, while the second returns continuous estimates. In the first group, the detected face is assigned to one of many discrete poses; usually the pose ranges are quantized by $15^{\circ }$. The main shortcomings of the approaches belonging to the discrete group are that they could not be used for head gesture recognition besides their fixed quantization error. The approaches developed by Ma *et al*. [14], Dahmane *et al*. [25], Zhu and Ramanan [16] use classification-based methods to classify each head image into one discrete pose. On the opposite, the approaches developed by Murphy-Chutorian *et al*. [12], Yang *et al*. [23] and Fanelli *et al*. [22] use regression-based methods to provide a continuous estimate of the head pose. Following the second group, other approaches fit a general/personalized head model to person’s head data to return a continuous estimate of his head pose [18,21].

A summary of the aforementioned approaches, where each approach is described in terms of the three criteria: temporal dependency; data source; and pose estimate continuity, are shown in Table 1.

**Table 1:** A summary of the state-of-the-art approaches. Each approach is described in terms of three criteria, its temporal-dependency, data source and the pose estimate continuity.

Approach	Temporal Dependency	Data Source	Estimation Continuity
Murphy-Chutorian *et al*. [12]	Frame-based	RGB	Continuous
Gurbuz *et al*. [17]	Frame-based	Stereo camera	Continuous
Jimenez *et al*. [18]	Temporal-dependent	Stereo camera	Continuous
Tu *et al*. [19]	Temporal-dependent	RGB	Continuous
Ruddarraju *et al*. [20]	Temporal-dependent	Multiple camera	Continuous
Niese *et al*. [21]	Temporal-dependent	Depth	Continuous
Fanelli *et al*. [22]	Frame-based	Depth	Continuous
Yang *et al*. [23]	Frame-based	Depth + RGB	Continuous
Buddharaju *et al*. [24]	Frame-based	Thermal image	Discrete
Ma *et al*. [14]	Frame-based	RGB	Discrete
Dahmane *et al*. [25]	Frame-based	RGB	Discrete
Zhu and Ramanan [16]	Frame-based	RGB	Discrete
The proposed approach	Frame-based	Depth + RGB	Continuous

Temporal-dependent approaches strongly rely on the initializing step, where most of them assume that the tracking starts from the frontal pose with approximately zero rotation angles. However, this assumption does not always hold true in real scenarios and would cause a fixed offset error. Some of these approaches employ the frontal model of the Viola and Jones (VJ) face detector [26] to start the tracking with zero rotation angles; however, this detector is capable of detecting faces across a wide range of poses ($\pm 30^{\circ }$ pitch, $\pm 40^{\circ }$ yaw, $\pm 20^{\circ }$ roll). To this end, we dedicate Section 3.1 to investigate the range of poses detected by the frontal model and to estimate the head pose by applying the proposed approach on top of it.

In this work, we propose a hybrid source approach for estimating the head pose exploiting the Kinect sensor. The face is detected in the gray image (transformed from the color image), while the features are extracted from both gray and depth images. To cover a wider range of head poses, we exploit both frontal and profile models for the face detection. Throughout this work, the VJ face detector is referring to the frontal and profile models that are available from the OpenCV library.

The contribution of this work can be summarized as follows.

- Investigating the range of poses supported by the frontal model of the VJ face detector.
- Providing a pose estimation approach working on top of the frontal and profile models of the face detector.
- Adopting three texture feature types for the task of pose estimation and presenting a fair comparison between them in terms of estimation accuracy and computation time.
- Introducing a new straightforward feature descriptor, extracted from the depth image, which provides competitive estimation results with a lower computation time.
- Exploiting the depth information offered by the Kinect sensor to enhance the state-of-the-art pose estimation accuracy.

The remainder of this work is organized as follows. In Section 2, we describe the proposed approach for the head pose estimation in terms of face detection, feature extraction and machine learning. A comprehensive evaluation of the proposed approach, including a comparison to state-of-the-art methods and presenting the benefits of utilizing depth data for the frame-based estimation, is provided in Section 3. A summary concludes the paper in Section 4.

## 2. The Proposed Approach

**Figure 2:**
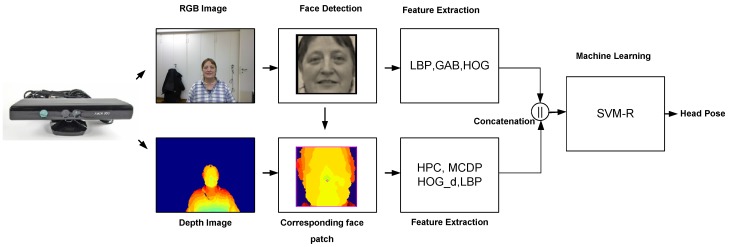
An overview of the proposed approach to estimate the human head pose. The Kinect sensor provides two sources of data: the RGB (color) image with its equivalent depth image. The face is located in the color image; then, several features are extracted from the face patches in both the color and depth image. These features are fed into SVM-R to infer a continuous estimate of the head pose.

Instead of using conventional 2D color cameras, the research community is using nowadays current RGBD sensor technology, which provides depth information besides the 2D color images. Kinect sensor type was launched worldwide in November 2010, which was the first time that computer vision played a pivotal role in a mass market [27]. With the Kinect sensor, you can have high-resolution depth sensing at a consumer price. By exploiting the depth data, we can overcome many traditional problems, such as: separating foreground from background pixels, unknown object scales and some lighting issues. In this work, we propose an approach for a frame-based head pose estimation, exploiting both data types (2D color image and depth data) offered by the Kinect, as shown in Figure 2. First, the face is automatically located in the 2D color image. Then, we extract different feature types from the detected face patch in the 2D color image and its corresponding 3D point cloud. These features encode the spatial distribution of the face texture over a box enclosing the detected face. Additionally, they encode the depth variation all over the face. Finally, the extracted features are concatenated to build a feature vector passed to support vector machine regressors (SVM-R) to return a continuous estimate of the head pose. Two experiments were conducted on a benchmark database. We investigated the accuracy of head pose estimators built using several concatenations of feature types besides using each feature type separately. Comparison to state-of-the-art approaches is discussed, as well. In what follows, the face detection, feature extraction and employed machine learning algorithm will be explained in detail.

### 2.1. Face Detection

**Figure 3:**
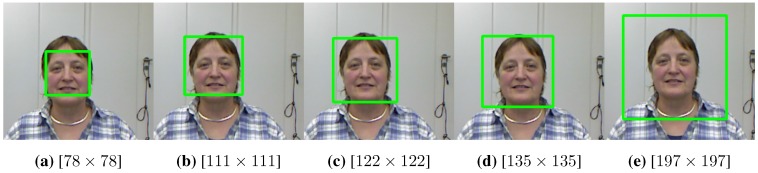
Different potential face detections by applying the Viola and Jones (VJ) face detector [26] to the same image with various parameters. The size of the returned box is shown beneath each sub-image. The image is taken from the Biwi database.

In this work, we employ the VJ approach to locate the face inside the 2D grayscale image (transformed from the RGB image). To detect faces inside an image using the OpenCV implementation [28], a sliding window shifts pixel-by-pixel, scanning the whole image across various scales for potential faces. Those window scales are parametrized by the minimum search size, maximum search size, and step scale factor. Each face outside the selected scales will be ignored. Then, the positive overlapping windows that passed the minimum neighboring threshold are merged through averaging to produce the final detection results. Using different setup values for the aforementioned parameters can cause inconsistent face cropping. Consequently, irregular detections are inadequate to be direct inputs into any training procedure. Figure 3 shows several inconsistent detections resulting from applying the VJ face detector to the same image each time with different parameters. In a similar way, scanning images that contain faces of different scales using the VJ detector with fix parameters results in inconsistent cropping patches.

**Figure 4:**
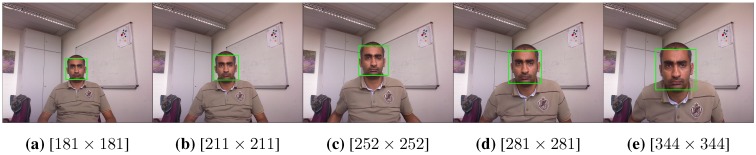
Using the VJ face detector to perform a two-stage search for the face. The face is consistently cropped at different scales. The size of the returned box is shown beneath each sub-image. The images are captured in our lab with a Kinect sensor at the Super Extended Graphics Array (SXGA) resolution (1280×1024).

To cope with this issue (inconsistent cropping), we apply the face detector two times. The first time, we perform a coarse localization where the margin between the minimum and maximum search size for the face bounding box is bigger and spans all of the potential face scales. Additionally, we use a relatively large step scale factor (1.5) and a lower minimum neighboring threshold (three). The second time, we perform a fine localization, where the minimum and maximum search sizes are taken by 200% and 70% of the face size detected in the first stage; the neighboring threshold is larger (six), and the step scale factor is lower (1.03). The second localization process is faster, since we narrow the search region to the area surrounding the detected face from the coarse localization. The returning box from the fine search is then considered for the feature extraction stage. As the final return face box by the VJ approach is an averaging of all overlapping detections, by performing the fine search, we guarantee the existence of similar detections invariant to the face scale; this then leads to a similar consistent cropping. The fine search improves the cropping consistency, not the detection rate, hence a false detection in the first search could not be corrected in the second search. The two-stage search results are shown in Figure 4, where the same face is consistently cropped at different scales. To have a computationally feasible approach, we are not using any further registration algorithm for the detected face.

Building a face pose estimator on top of this face detector gains importance from the wide spread use of it in various applications, such as facial expression recognition [29,30] and human age recognition [31]. Therefore, the proposed approach can be used to enhance the performance of those approaches for non-frontal faces. Additionally, optimized implementations of the VJ face detector are publicly available. For example, the OpenCV library is offering a GPU implementation for it. Sharma *et al*. [32] showed the feasibility of building the VJ face detector in real time.

### 2.2. Feature Extraction

As the facial geometry and appearance vary significantly across the head pose, exploiting mixed feature types that encode those characteristics is the way to infer the head pose. To this end, we extract several feature types from the detected face patch in the gray image and its corresponding patch in the depth image. Those features can be divided into two groups based on their data source as follows.

#### 2.2.1. Appearance-Based Features

Three appearance-based feature types are employed here to tackle the head pose estimation from cropped faces in the grayscale images. Those features have shown a great capability to encode both local and global textures of pictured patches.

**Figure 5:**
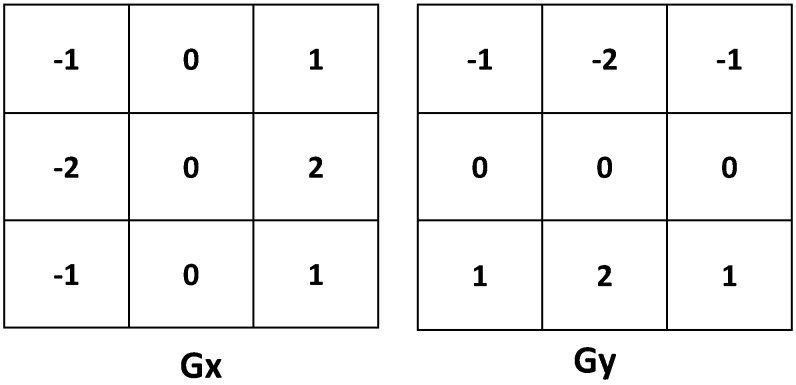
Horizontal (Gx) and vertical (Gy) Sobel’s kernels used to calculate the image gradient, a step to derive the HOG feature vector.

- Gabor filter-based features (GAB): This type of feature has functional similarity to certain cells in the human primary visual cortex; additionally, it has a spatial frequency localization property. It is defined as a Gaussian kernel modulated by a sinusoidal wave as follows:
  (1) $$\begin{array}{ccc} g(x,y;\lambda ,\theta ,\sigma_{x},\sigma_{y}) & = & \exp\left\{-\frac{1}{2}\left(\frac{{\overset{´}{x}}^{2}}{\sigma_{x}^{2}}+\frac{{\overset{´}{y}}^{2}}{\sigma_{y}^{2}}\right)\right\}\\ & & \times \exp\left\{i(2\pi \frac{\overset{´}{x}}{\lambda })\right\} \end{array}$$
  where *λ* is the frequency (in pixel) and *θ* is the orientation of the sinusoidal function. $\sigma_{x}$ and $\sigma_{y}$ are the standard deviations along the x- and the y-axis, and $\overset{´}{x}=x\cos\theta +y\sin\theta$, $\overset{´}{y}=-x\sin\theta +y\cos\theta$. Obviously, the real and/or imaginary components of the filter can be derived from Equation (1) and used alone or together. For our approach, we utilize three scales of Gabor kernels, each generated with two different values for *λ*, seven for *θ* and two for each *σ*. After applying each kernel to the scaled 100×100 pixel detected face patch, we divide the resulting patch into smaller 10×10 pixel cells. Then, we extract the median value of each cell and normalize these values to generate a kernel feature vector. Finally, we concatenate the vectors from all kernels to produce the *GAB* feature vector of a length of 2800.
- Local binary pattern features (LBP): The LBP was originally introduced by Ojala *et al*. [33], where each pixel is labeled by thresholding its value with neighborhood pixel values; then, the results are combined into a binary number. Besides its computational simplicity and illumination invariance, LBP encodes different texture primitives, such as: spot, edge and corner. Let $f_{c}$ and $f_{p}$ denote the pixel values at the center and neighboring pixels, respectively, where p=0,...,7. Then, each binary value B(p) of the $LBP_{v}$ is calculated as:
  (2) $$\begin{array}{c} B\left(p\right)=\left\{\begin{array}{cc} 1 & \mathrm{if}\;\;f_{c}>f_{p}\\ 0 & \mathrm{otherwise} \end{array}\right. \end{array}$$
  Next, we assign a binomial factor $2^{p}$ for each B(p) to get $LBP_{v}$ as follows:
  (3) $$LBP_{v}=\sum_{p=0}^{7}B\left(p\right)\times 2^{p}$$
  Throughout this work, we calculate the $LBP_{v}$ (as in Equation (3)) for each pixel of the scaled 200×200 pixel detected face patch. Then, we divide the face patch into smaller 10×10 pixel cells. Next, we calculate an eight-bin histogram for each cell. Finally, those histograms are concatenated to form the LBP feature vector of a length of 3200.
- Histogram of oriented gradients (HOG): HOG was originally introduced by Dalal and Triggs [34], where it was employed for pedestrian detection. First of all, the image is divided into small spatial regions called cells. For each pixel in the cell, the orientation gradients are calculated. Then, for each cell, a 1D histogram of orientation is formed, where each pixel vote is weighted by its magnitude. For the purpose of having better invariance to illumination and shadowing, normalization over larger spatial regions is performed before extracting the final descriptor, where those larger regions may also overlap. In this work, we divide the scaled 200×200 pixel detected head patch into smaller 20×20 pixel cells. The *x*- and *y*-gradients of the head patch are calculated with the help of horizontal (Gx) and vertical (Gy) Sobel kernels, which are shown in Figure 5. Then, we calculate an eight-bin orientation histogram for each 40×40 pixel block region, where each block region comprises four cells. A block spacing stride of 20 pixels is used. The final feature vector is of a length of 2592.

#### 2.2.2. Depth-Based Features

Depth-based features are defined as those features extracted from the matching face patch in the depth image. Here, we encode the 3D shape information of the face. As the face shape varies across the head pose, those features are distinctive for the head pose inference, as well as the appearance-based features.

**Figure 6:**
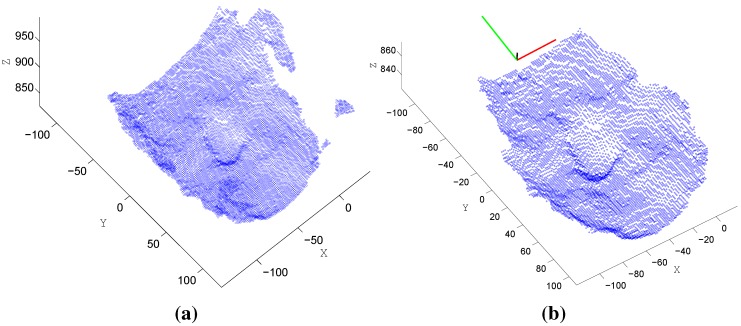
Extracting the head point cloud features (HPC). (**a**) The recovered 3D points of the captured face; (**b**) The filtered points by Equation (4) and the eigenvector direction shown on the top of the sub-image. *X*, *Y*, *Z* represent the real coordinates in mm.

- Head point cloud features (HPC): This feature type encodes roughly the orientations of the face point cloud, which is much closer to the head pose. First, we retrieve the point cloud of the matching head patch (HP) in the depth image. Only a certain depth range (DR) is allowed in order to eliminate any surrounding objects. In this feature type, we allow only the points that are not far by more than 50 mm from the closest head point to the camera. Using a simple pinhole camera model, for a pixel $\left(x_{i},y_{i}\right)$, we get its corresponding 3D point $p_{i}$ as follows.
  (4) $$\begin{array}{c} p_{i}=\left(\begin{array}{c} \frac{-z_{i}\left(x_{i}-c_{x}\right)}{f_{x}}\\ \frac{-z_{i}\left(y_{i}-c_{y}\right)}{f_{y}}\\ z_{i} \end{array}\right),\;\;\;\;\;\left(x_{i},y_{i}\right)\in \mathrm{HP},z_{i}\leq \mathrm{DR} \end{array}$$
  $z_{i}$ denotes the corresponding depth value for the pixel $\left(x_{i},y_{i}\right)$. $\left(c_{x},c_{y}\right)$ is the principal point, usually at the image center. Here, the image distortions are ignored due to their negligible effect using the Kinect sensor. $f_{x}$, $f_{y}$ are the focal lengths expressed in pixel units. We deal with the 3D points within each image patch as a random variable p with *n* samples satisfying Equation (4). Next, we calculate the covariance matrix over all points as follows.
  (5) $$\Sigma =\text{E}\left[\left(p-\text{E}[p]\right){\left(p-\text{E}[p]\right)}^{\text{T}}\right]$$
  where E(x) is the expected value (or mean) of *x*. ${\left[\;\right]}^{\text{T}}$ denotes the matrix/vector conjugate transpose. Following this, we apply the singular value decomposition (SVD) to the covariance matrix Σ, obtained by Equation (5), to be written as follows.
  (6) $$\Sigma =USV^{\text{T}}$$
  U,S,V are matrices of size 3×3. U and V are unitary matrices. S is a diagonal matrix with diagonal entries $\left(\sqrt{{\tilde{\lambda }}_{i}}\right)$ equal to the square root of eigenvalues from $\Sigma \Sigma^{\text{T}}$. Additionally, the eigenvectors of $\Sigma \Sigma^{\text{T}}$ make up the columns of V. The eigenvectors describe the orthogonal principal directions of the point (**P**) variation from their mean, with corresponding standard deviation $\left(\sqrt{{\tilde{\lambda }}_{i}}\right)$. Finally, we concatenate the eigenvectors and use them as a feature vector of a length of nine encoding the point cloud general orientation. Figure 6 shows the steps of extracting this feature type, where the whole 3D points of the captured face are depicted in Figure 6a and the filtered points (according to Equation (4)) along with the extracted eigenvectors in Figure 6b.
- Multi-scale comparative depth patches (MCDP): Except the aforementioned HPC features, all other depth-based features consider the spatial distribution of the facial depth variation as a valuable cue for the head pose estimation. Here, we divide the face patch into smaller, equally-sized cells, four times each with a different cell scale. Then, for each scale, we calculate the average depth value of each cell and normalize these values for all cells of the same scale. Finally, we concatenate all normalized values from the four scales to produce a final MCDP descriptor vector of a length of 512. The HPC and MCDP descriptors are introduced in this work for the first time. These two feature types are straightforward. In HPC, we encode the global orientations of the head point cloud, and in MCDP, we encode the spatial structure of the 3D facial data. Those cues are considered valuable to infer the head pose.
- Depth geometry features: To encode the face depth geometry along with its spatial structure, we apply the previously-mentioned HOG and LBP on the depth face patch. We denote the resulting descriptors as ${\mathrm{HOG}}_{d}$ and ${\mathrm{LBP}}_{d}$.

The parameters of each descriptor, such as: cell size; histogram bins; and used scales, are determined through cross-validation experiments carried out on the training sets.

### 2.3. Machine Learning Approach

We prefer employing a regression-based machine learning approach rather than a discrete classification-based one to produce a continuous estimate of the head pose. This would give our approach the advantage to be used in head gesture recognition and to avoid the fixed quantization error. The Support Vector Machine (SVM) is well known for its generalization capability and avoiding overfitting in the multi-class classification and regression, as well [35]. Let the input-output training pairs be denoted by $(x_{i},y_{i})\;$ (i=1,⋯,N), where $x_{i}$ is the *i*-th *m*-dimensional training sample (input feature vector) and $y_{i}$ is the *i*-th scalar output value (pose value). In SVM regression, the input vector is mapped into higher dimensional feature space where the optimal hyperplane is given by:

(7) $$f\left(x\right)=w^{\text{T}}\phi \left(x\right)+b$$

w is the *l*-dimensional weight vector; ϕ(x) is the mapping function that maps x into the *l*-dimensional feature space; and *b* denotes the bias term. A piecewise linear function is used as an error function, such that:

(8) $$\begin{array}{c} E_{r}\left(y-f\left(x\right)\right)=\left\{\begin{array}{cc} 0 & \mathrm{for}\;\;\;|y-f(x)|\leq \varepsilon \\ |y-f(x)|-\varepsilon & \mathrm{otherwise} \end{array}\right. \end{array}$$

**Figure 7:**
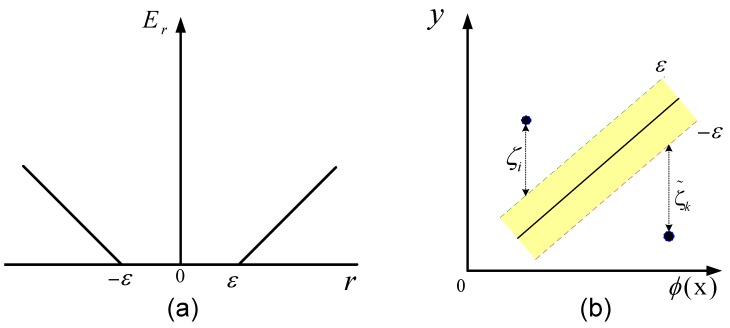
(**a**) The SVM error function where *r* is the residual (r=y-f(x)); (**b**) *ε*-insensitive zone.

As shown in Figure 7, the ideal estimation is realized when the absolute residual is within *ε* (*ε* insensitive zone), namely:

(9) |y-f(x)|≤ε

For feasible solutions, non-negative slack variables $\left(\zeta ,\tilde{\zeta }\right)$ are introduced for the training samples that are outside the tube of radius *ε*.

(10) $$\begin{array}{c} \zeta_{i}=\left\{\begin{array}{cc} 0 & \mathrm{for}\;\;\;y-f(x)-\varepsilon \leq 0\\ y-f(x)-\varepsilon & \mathrm{otherwise} \end{array}\right. \end{array}$$

(11) $$\begin{array}{c} {\tilde{\zeta }}_{i}=\left\{\begin{array}{cc} 0 & \mathrm{for}\;\;\;y-f(x)+\varepsilon \geq 0\\ -(y-f(x))-\varepsilon & \mathrm{otherwise} \end{array}\right. \end{array}$$

Minimizing ||w|| leads to maximizing the margin; the margin here means the farthest distance from the hyperplane to the training samples that are inside the *ε*-tube. As the margin increases, the generalization probability is increasing. Finally, the SVM regression problem is formulated as follows.

(12) $$\begin{array}{cc} \text{Minimize} & \;\frac{1}{2}{∥w∥}^{2}+C\sum_{i=1}^{N}\left(\zeta_{i}+{\tilde{\zeta }}_{i}\right)\\ \text{subject}\;\text{to} & \;y_{i}-w^{\text{T}}\phi \left(x_{i}\right)-b\leq \varepsilon +\zeta_{i}\\ & \;w^{\text{T}}\phi \left(x_{i}\right)+b-y_{i}\leq \varepsilon +\tilde{\zeta_{i}}\\ & \;\zeta_{i}\geq 0,\;{\tilde{\zeta }}_{i}\geq 0,\;\;\;\;\forall i \end{array}$$

where *C* is the margin parameter defining the trade-off between the margin value and the estimation error of the training data. The regression problem in Equation (12) is an optimization problem, which would be solved with the help of quadratic programming techniques. To estimate the head pose in this work, we built three regressors, each corresponding to one rotation angle. The parameters of each regressor are chosen using grid search with cross-validation. For more reading about the employed SVM-R, the readers are referred to [35,36].

## 3. Experimental Results

Two experiments were carried out to evaluate our proposed approach. First, we investigate the detection capability of the VJ frontal model across the head poses. Additionally, we present the pose estimation accuracy by applying our approach on top of it. In the second experiment, we employ both frontal and profile models for the face detection to cover a wider range of poses, where a comparison with state-of-the-art approaches is presented, as well. We end this section by presenting a comparison of the exploited features in terms of time consuming to complement the comparisons of the pose estimation accuracy that were provided by the two experiments. Throughout this section, only true positive detections, defined as the detections that overlap with the ground truth location of the face, are evaluated.

For our evaluations and comparisons, we employed the Biwi database, which is publicly available. This database was produced by Fanelli *et al*. [22]. For each frame instance, they provide color and depth images stemming from a Kinect sensor. The database comprises 24 sequences of 20 different people (14 men and six women, four wearing glasses), recorded while sitting about one meter away from the sensor. Each subject rotated his head spanning all possible ranges of the three rotation angles (pitch, yaw, roll). With the help of a personalized template, each subject’s sequence of head rotations was tracked using ICP to provide a ground truth estimate for each frame. The database contains 15,678 frames with rotation angles ranging around $\pm 75^{\circ }$ for yaw, $\pm 60^{\circ }$ for pitch and $\pm 40^{\circ }$ for roll. The images are in a VGA resolution (640×480 pixel), where the average cropped face size is 95×95 pixel. We select this database as it provides simultaneous color and depth images besides its accurate methodology for calculating continuous ground truth values.

### 3.1. Experiment 1: Frontal Model Analysis

**Figure 8:**
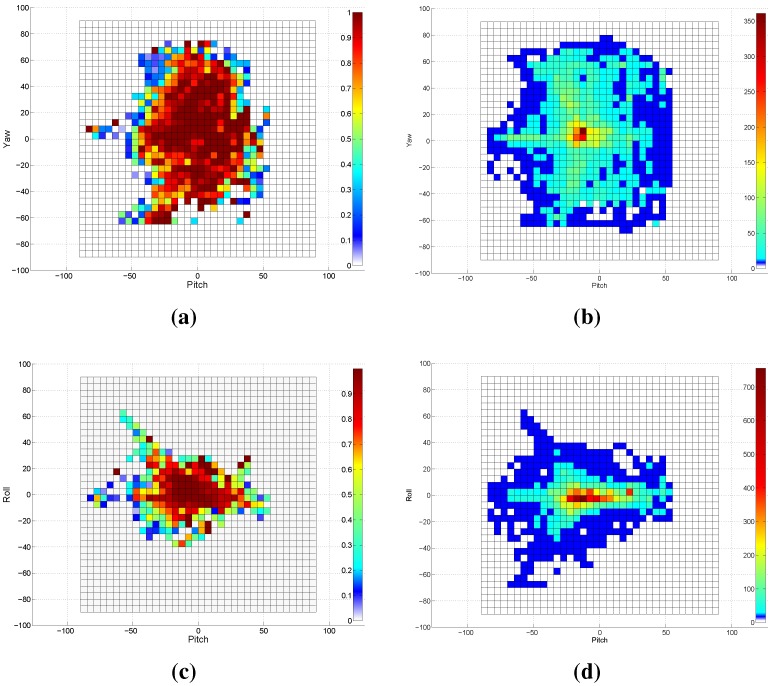
The results of applying the frontal model of the VJ face detector on the Biwi Kinect Head Pose Database. (**a**) The detection rate across yaw and pitch angles in degrees; (**b**) This subfigure is complementing (**a**) by showing the number of samples for each yaw-pitch grid; (**c**) The detection rate across roll and pitch angles; (**d**) This subfigure is complementing (**c**) by showing the number of samples for each roll-pitch grid.

In this experiment, we investigate the detection capability of the VJ frontal model across the head poses. We applied this face detector to the entire Biwi database (15,678 images). The face was detected in approximately 75% of the entire database; all detected face patches will participate in the evaluation of our proposed approach. Figure 8 summarizes the obtained results by this experiment. Figure 8a shows the grid detection rate across the pitch and yaw rotation angles, where the detection rate measures the proportion of the images in which the human face was detected to the entire images of the underlying grid. To complement Figure 8a, we provide the total number of Biwi images across the yaw and pitch angles in Figure 8b. In a similar way, the results are depicted for the pitch roll axes in Figure 8c,d. The lower pose angles ($\pm 15^{\circ }$ pitch, $\pm 15^{\circ }$ yaw, $\pm 10^{\circ }$ roll) are represented in the database with more samples compared to the other rotation angles. From Figure 8, we can deduce that the frontal model of the VJ approach is capable of detecting faces of poses spanning $\pm 30^{\circ }$ pitch, $\pm 20^{\circ }$ roll, $\pm 40^{\circ }$ yaw, with an 80% detection rate at the minimum. The poses beyond those ranges could be detected with lower detection rates.

We apply the proposed approach to estimate the head pose on top of the frontal model, where the training and evaluation phases are limited to the frames in which the human face is detected. The head patches and their corresponding depth images are fed into the feature extractor, as shown in Section 2. Then, the head pose is estimated by applying the pre-trained SVM-R to the extracted feature vector. Table 2 summarizes the results of employing single and concatenated feature types for the head pose estimation. The error is defined as the absolute value of the difference between the ground truth angle and its corresponding estimated one. For each experiment, we reported the mean and standard deviation of the estimation error for each rotation angle. Similar to Fanelli *et al*. [22], we divided the database into training and testing sets of 18 and two subjects (leave two out cross-validation), respectively. Samples of the same person do not exist in both training and testing sets.

**Table 2:** The mean/standard deviation of the absolute error for each estimated head pose angle. The feature column indicates the used single feature type or concatenation of more than one. This experiment was carried out on the Biwi database. LBP, local binary pattern; GAB, Gabor; MCDP, multi-scale comparative depth patches.

Feature	Pitch Error ${(}^{\circ })$	Yaw Error ${(}^{\circ })$	Roll Error ${(}^{\circ })$
${\mathrm{LBP}}_{d}$	8.9 / 8.5	8.8 / 8.9	4.8 / 5.9
LBP	12.4 / 10.5	12.6 / 13.5	4.7 / 5.3
GAB	9.8 / 8.5	7.6 / 7.5	4.4 / 4.6
HOG	6.9 / 6.8	6.3 / 7.7	3.1 / 4.2
${\mathrm{HOG}}_{d}$	4.9 / 5.8	6.1 /6.8	3.8 / 4.7
$\mathrm{HOG}+{\mathrm{HOG}}_{d}$	4.3 / 5.5	5.5 / 6.5	3.0 / 4.3
$\mathrm{LBP}+{\mathrm{LBP}}_{d}$	8.6 / 8.7	8.8 /8.9	4.6 / 5.8
HPC+MCDP	5.3 / 5.4	5.6 /5.4	4.3 / 4.8
$\mathrm{HOG}+{\mathrm{HOG}}_{d}+\mathrm{HPC}+\mathrm{MCDP}$	4.0 / 5.1	4.3 /5.4	2.9 / 4.2

A number of points can be drawn from the obtained results as follows. The pose estimation is more accurate when we employ the same feature type on the depth image rather than on the color image. For example, we achieved a pitch mean error of $8.9^{\circ }$ with ${\mathrm{LBP}}_{d}$ compared to $12.4^{\circ }$ with LBP and $8.8^{\circ }$ *versus* $12.6^{\circ }$ for the yaw angle, whereas the estimation mean errors for the roll angle are equal using both ${\mathrm{LBP}}_{d}$ and LBP. In a similar way, ${\mathrm{HOG}}_{d}$ provides more accurate estimation compared to HOG for both pitch and yaw angles, while being slightly less accurate for roll angle. Regarding the appearance-based features, the most accurate pose estimation is achieved by HOG, where the pose mean errors are $6.9^{\circ },6.3^{\circ },3.1^{\circ }$ for pitch, yaw and roll, respectively, compared to $9.8^{\circ },7.6^{\circ },4.4^{\circ }$ achieved by GAB and even greater errors ($12.4^{\circ },12.6^{\circ },4.7^{\circ }$) by LBP. Regarding the depth-based features, the ${\mathrm{HOG}}_{d}$ provides more accurate pose estimations, which are slightly better than those using the newly-introduced descriptors: HPC+MCDP. On the other side, we got the greatest error in the estimated pose angles using ${\mathrm{LBP}}_{d}$. Concatenating depth-based and appearance-based features leads to an improvement in the estimation accuracy, as we can see that ${\mathrm{HOG}}_{d}+\mathrm{HOG}$ performed better than using them individually; and in a similar way, $\mathrm{LBP}+{\mathrm{LBP}}_{d}$ performed better than using them separately. Our most accurate estimations were obtained by concatenating the appearance-based feature HOG with ${\mathrm{HOG}}_{d}+\mathrm{HPC}+\mathrm{MCDP}$ depth-based features, where we estimate the pitch angle with $4.0^{\circ }$ as a mean error, yaw with $4.3^{\circ }$ and roll with $2.9^{\circ }$.

### 3.2. Experiment 2: Comparisons with the State-of-the-Art

**Figure 9:**
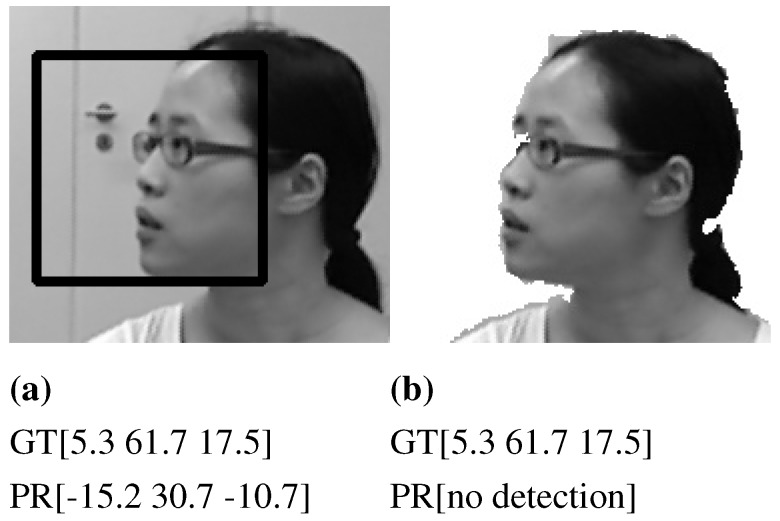
Sample of inconsistent face cropping due to the background texture. (**a**) Wrong face cropping using VJ frontal model; (**b**) The face with a white background, not detected using the frontal model. GT denotes the ground truth rotation angles (pitch, yaw, roll), and PR is the predicted angles.

**Figure 10:**
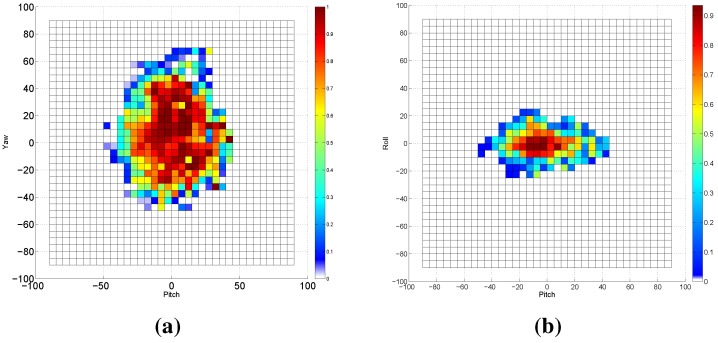
The results of applying the frontal model of the VJ face detector on the Biwi database with a white background. (**a**) The detection rate across yaw and pitch angles in degrees; (**b**) The detection rate across roll and pitch angles.

We have found that the frontal model detects profile faces when a complementary background exists. Those detections are wrongly cropped, resulting in greater pose estimation errors, as seen in Figure 9a where the predicted angles (PR) are far from the ground truth (GT) values. In order to avoid such detections, we whitened the background out with the help of the depth data, as shown in Figure 9b. Consequently, the detection rates drawn in Figure 8a,c are updated and depicted in Figure 10. It is clearly shown that the detection rate using the frontal model for faces in profile poses (faces with higher yaw angle) is decreased. However, the frontal model is still capable of detecting faces of poses ranging between $\pm 30^{\circ }$ pitch, $\pm 20^{\circ }$ roll and $\pm 40^{\circ }$ yaw with higher detection rates, which proves that the assumption of using the frontal VJ face detector to initialize a pose tracker with zero rotations is weak and does not hold true for many cases.

**Figure 11:**
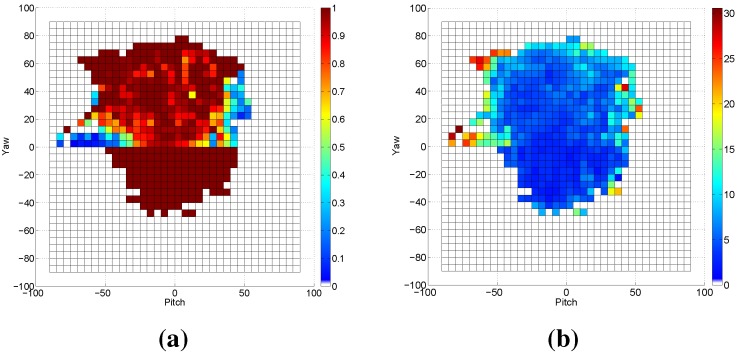
The results of applying the frontal and profile models of the VJ face detector on the Biwi database with a white background. (**a**) The detection rate across yaw and pitch angles in degrees; (**b**) The average error of the estimated angles across yaw and pitch angles.

**Figure 12:**
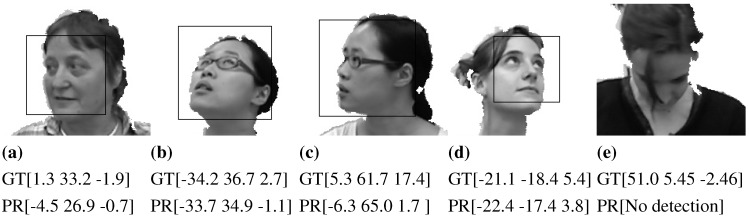
Samples of head pose estimations taken from the Biwi database, where a concatenation of $\mathrm{HOG}+{\mathrm{HOG}}_{d}+\mathrm{HPC}+\mathrm{MCDP}$ feature types is employed. GT denotes the ground truth rotation angles (pitch, yaw, roll), and PR is the predicted angles.

To cover a wider range of head poses in the database, we exploit both frontal and profile models for the face detection, where the profile model is applied when the frontal model fails to return a true positive detection. Figure 11a depicts the detection rate using both frontal and profile models. Obviously, the use of the profile model causes higher detection rates for the faces at significant yaw angles, reaching more than 95% in most grids. The faces with extreme pitch angles are still hard to detect, as shown in Figure 12e. In this experiment, a white background and leave two out cross-validation are employed. Table 3 summarizes the cross-validation of several concatenations along with reported results of two state-of-the-art approaches. A number of points can be drawn from the obtained results as follows. The pose estimation with features from the depth image is more accurate than that from the gray image; however, this advance in the accuracy is less than that in Table 2, as the depth data already affect the gray image by whiting the background out. In a similar way to Table 2, concatenating both feature types leads to more accurate estimates. The pose estimation using HOG features (or any concatenation involving it) is more accurate in comparison to the-state-of-art approaches. HPC+MCDP provides competitive estimation accuracy, outperforming the state-of-the-art results, as well. The concatenation of $\mathrm{HOG}+{\mathrm{HOG}}_{d}+\mathrm{HPC}+\mathrm{MCDP}$ provides the most accurate estimates, where the average errors are not exceeding $5.1^{\circ },4.6^{\circ },4.2^{\circ }$ for pitch, yaw and roll, respectively. The mean error of the estimated three angles, resulting from the use of $\mathrm{HOG}+{\mathrm{HOG}}_{d}+\mathrm{HPC}+\mathrm{MCDP}$ concatenation, is depicted in Figure 11b. Interestingly, the estimation is accurate for high yaw angles as for the low ones. On the other hand, the estimation error is increasing as the pitch angle gets high, which in most cases is due to false cropping. Those outperforming results can be attributed to many factors: (1) employing the most distinctive features ($\mathrm{HOG},{\mathrm{HOG}}_{d},\mathrm{HPC}+\mathrm{MCDP}$) for the head pose estimation; (2) employing the profile model of the face detector, which guarantees high accuracy in the profile cases as shown in Figure 12b,c; (3) the consistent face cropping using the proposed two-step search using the VJ face detector; (4) the generalizing capability of the exploited SVM regressors; (5) the parameters (of the feature extractors and regressors) estimation using grid search with cross-validation on the training set. Figure 12 shows samples of our cross-validation evaluation on the Biwi database, where Figure 12a,b are samples of the frontal model detection, while Figure 12c,d of the profile model. The face in Figure 12 cannot be detected by both models.

**Table 3:** The mean/standard deviation of the absolute error for each estimated head pose angle using several feature concatenations, along with reported results of two state-of-the-art approaches. The frontal and profile models of the VJ were applied on the Biwi database with a white background.

Algorithm	Pitch Error ${(}^{\circ })$	Yaw Error ${(}^{\circ })$	Roll Error ${(}^{\circ })$
${\mathrm{LBP}}_{d}$	10.9/9.8	9.1/7.8	6.4/6.1
LBP	11.3/10.5	9.7/8.5	7.0/6.7
GAB	11.2/10.9	8.5/7.1	6.1/6.2
HOG	7.4/7.2	6.1/5.7	4.5/4.9
${\mathrm{HOG}}_{d}$	7.1/8.2	5.9/6.3	5.3/5.7
$\mathrm{LBP}+{\mathrm{LBP}}_{d}$	8.9/9.1	7.9/7.2	6.3/5.9
$\mathrm{HOG}+{\mathrm{HOG}}_{d}$	6.3/6.1	5.4/5.3	4.3/4.2
HPC+MCDP	7.6/7.4	6.6/6.1	4.9/4.8
$\mathrm{HOG}+{\mathrm{HOG}}_{d}+\mathrm{HPC}+\mathrm{MCDP}$	5.12/5.3	4.6/4.5	4.2/4.1
Fanelli *et al*. [22]	8.5/9.9	8.9/13.0	7.9/8.3
Yang *et al*. [23]	9.12/7.40	8.92/8.27	7.42/4.90

### 3.4. Processing Time

In this work, we build our pose estimation on top of the VJ face detector. One of the advantages of using this detector is the publicly available optimized codes for it. Sharma *et al*. [32] proved that a speed of 45 frames per second could be reached by processing a 640×480 pixel image with CUDA.

In this section, we compare the pose estimation speed, in terms of feature extraction and regression times, for the proposed feature types. This experiment is carried out on the Biwi database using Intel quad Core 2.33 GHZ, 8 GB RAM, under the Windows environment. We did not utilize the parallel programming possibility or the GPU for the feature extraction and regression. Table 4 shows the time required to extract and employ the regression models for each feature type. These time values were recorded when we used each feature type alone, assuming concatenated cases will consume the sum of all contained feature times.

**Table 4:** The process time of the pose estimation in terms of feature extraction and regression times. To get an intuitive meaning, the times are presented as s/frame per second (s/fps).

Feature	Extraction Time (s/fps)	Regression Time (s/fps)
LBP	0.011/90	0.016/60
GAB	0.1/10	0.0142/70
HOG	0.010/100	0.0149/67
HPC+MCDP	0.003/300	0.0025/400

Extracting the GAB features is the most time-consuming process among other feature types. However, the pose estimation accuracy using GAB is not the best. HOG and LBP are extracted and classified at approximately the same time. The most interesting result is that the depth features HPC+MCDP, which are introduced by this work for the first time, are extracted and classified at a higher speed. Furthermore, they provide competitive estimating results, as shown in Table 2 and Table 3. Concatenating several feature types will definitely enhance the estimation accuracy, but at the cost of more processing time.

Since we apply our feature extraction and regression to a single detected face and the face detector is working in our lab at 45 fps on NVIDIA GeForce GTX 780 (640×480, all potential scales), it is possible to achieve a real-time frame rate for the pose estimation with the current implementation. Improving the time efficiency of the proposed approach can be achieved using parallel programming in extracting the features and in employing the regression models or using faster regression algorithms (or different parameters for the current one), which can be at the cost of lower estimation accuracy.

## 4. Conclusions and Future Work

Head pose estimation is crucial for many advanced facial analysis tasks in various computer vision systems, such as: facial expression recognition, head gesture recognition, gaze recognition and driver monitoring. In this work, we proposed a frame-based approach to provide a continuous estimate of the head pose, utilizing both depth and color images offered by the Kinect sensor. Our approach was built on top of the Haar-like face detector, which is widely employed due to its efficient implementation and proper performance. The frontal model is not limited to zero rotation angles, but rather can detect faces of poses spanning $\pm 30^{\circ }$ pitch, $\pm 20^{\circ }$ roll, $\pm 40^{\circ }$ yaw with a high detection rate. However, to cover a wider range of poses, we exploited the frontal and profile models of the face detector. We adopted three texture feature types for the pose estimation task, presenting a fair comparison between them in terms of estimation accuracy and computation time. Additionally, we introduced straightforward depth-based feature types (HPC+MCDP) that provide competitive estimation accuracy with a lower computation time. With a concatenation of $\mathrm{HOG}+{\mathrm{HOG}}_{d}+\mathrm{HPC}+\mathrm{MCDP}$, we outperformed the pose estimation accuracy obtained by the state-of-the-art approaches. Utilizing the estimated head pose for head gesture and facial expression recognition is the next step in our research.
